# Extrapulmonary tuberculosis, human immunodeficiency virus, and foreign birth in North Carolina, 1993 – 2006

**DOI:** 10.1186/1471-2458-8-107

**Published:** 2008-04-04

**Authors:** Aaron M Kipp, Jason E Stout, Carol Dukes Hamilton, Annelies Van Rie

**Affiliations:** 1 Department of Epidemiology, University of North Carolina, Chapel Hill, North Carolina, USA; 2 Division of Infectious Diseases and International Health, Duke University Medical Center, Durham, North Carolina, USA

## Abstract

**Background:**

The proportion of extrapulmonary tuberculosis (EPTB) reported in the United States has been gradually increasing. HIV infection and foreign birth are increasingly associated with tuberculosis and understanding their effect on the clinical presentation of tuberculosis is important.

**Methods:**

Case-control study of 6,124 persons with tuberculosis reported to the North Carolina Division of Public health from January 1, 1993 to December 31, 2006. Multivariate logistic regression was used to obtain adjusted odds ratios measuring the associations of foreign birth region and US born race/ethnicity, by HIV status, with EPTB.

**Results:**

Among all patients with tuberculosis, 1,366 (22.3%) had EPTB, 563 (9.2%) were HIV co-infected, and 1,299 (21.2%) were foreign born. Among HIV negative patients, EPTB was associated with being foreign born (adjusted ORs 1.36 to 5.09, depending on region of birth) and with being US born, Black/African American (OR 1.84; 95% CI 1.42, 2.39). Among HIV infected patients, EPTB was associated with being US born, Black/African American (OR 2.60; 95% CI 1.83, 3.71) and with foreign birth in the Americas (OR 5.12; 95% CI 2.84, 9.23).

**Conclusion:**

Foreign born tuberculosis cases were more likely to have EPTB than US born tuberculosis cases, even in the absence of HIV infection. Increasing proportions of foreign born and HIV-attributable tuberculosis cases in the United States will likely result in a sustained burden of EPTB. Further research is needed to explore why the occurrence and type of EPTB differs by region of birth and whether host genetic and/or bacterial variation can explain these differences in EPTB.

## Background

**Figure 1:**
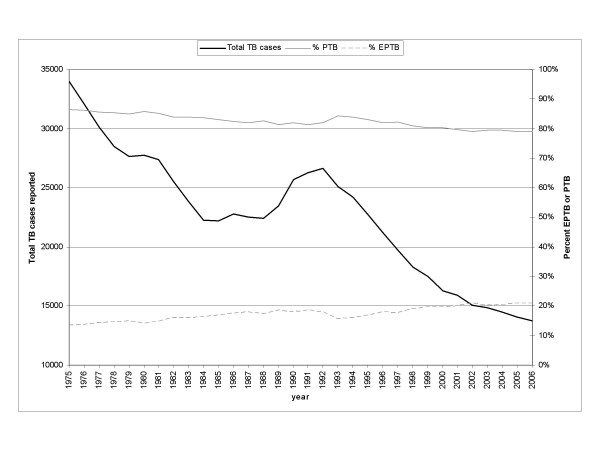
Reported tuberculosis cases and proportion by site of disease, United States, 1975–2006. (Source: References [3–6]).

The incidence of tuberculosis (TB) in the United States (US) has been declining over the past decades except for a resurgence from 1985 to 1992 (Figure 1) [1,2]. In 2006, the number of TB cases in the US reached an all-time low with 13,779 new cases, corresponding to an incidence of 4.6 cases/100,000 persons [3]. The decline in extrapulmonary TB (EPTB) has not been as great as for pulmonary TB (PTB). Consequently, the proportion of EPTB cases has increased from 13.5% of all reported TB cases in 1975 to 21.0% in 2006 (Figure 1) [3-6]. This relative increase may be an underestimate due to recent changes in case definitions, as cases with concomitant pulmonary disease or miliary disease are now counted as PTB cases [3-6].

Several studies have observed that the proportion of EPTB is higher among HIV co-infected individuals [7-11] and foreign born immigrants [8,9,12,13]. The latter population currently accounts for over half of all TB cases in the US [3].

Few studies have quantified the independent effect of HIV and foreign birth on EPTB and none have analyzed their joint association with EPTB. In this study, we investigated the association of HIV and foreign birth location, both individually and jointly, with the occurrence of EPTB among reported TB cases in North Carolina from 1993 to 2006.

## Methods

We analyzed all verified TB cases in the North Carolina TB registry that were reported to the Division of Public Health from January 1, 1993 through December 31, 2006. Cases were either laboratory, clinical, or provider verified using standard definitions from the Centers for Disease Control and Prevention (CDC) [3]. Demographic information, TB risk factors, disease presentation, and diagnostic and treatment information on each case were collected using the CDC's standardized Report of Verified Case of Tuberculosis form and entered into the North Carolina registry.

### Variables used

The outcome of interest was EPTB, defined as any verified TB case whose site(s) of disease was not recorded as "Pulmonary". Individuals with concomitant PTB and EPTB were excluded due to insufficient numbers and because we aimed to analyze the effect of HIV and foreign birth region on exclusive EPTB.

Exposures of interest included HIV infection, foreign birth, region of birth, and US born race/ethnicity. HIV status was defined as positive, negative, or unknown. Foreign birth was defined as being born outside of the US or its territories [3]. US and foreign born TB cases were categorized by race/ethnicity and region of birth, respectively, to account for differences in TB rates. US TB cases were categorized as White/Caucasian, Black/African American, Hispanic, or Other (Asian, American Indian, Native Alaskan, or Pacific Islander). Foreign born cases were classified as African, American (Central and South America and Caribbean), European (including the Middle East and Russia), Indian (including Pakistan), Southeast Asian, and East Asian (further detail provided in the accompanying Appendix).

To allow for a detailed analysis of the association of EPTB with HIV and foreign birth, both individually and jointly, HIV status and race/ethnicity or birth region variables were combined into exclusive categories: HIV positive and foreign born by region, HIV positive and US born by race/ethnicity, HIV negative and foreign born by region, and HIV negative and US born by race ethnicity. US born, White/Caucasian TB cases who were HIV negative served as the common referent group.

### Statistical Analysis

Crude odds ratios (OR) and ORs from logistic regression were used to measure the association between each exposure category and EPTB. A final model was derived using backward elimination [14,15]. Further interactions beyond HIV infection by race/ethnicity or birth region were not considered. All covariates were assessed for confounding. If the OR changed by more than 10% when a covariate was removed from the model, that covariate was considered a confounder and retained in the model [16]. Finally, a year variable was included in the model to account for increasing implementation of routine HIV testing during the study period.

All analyses were performed using SAS version 9.1.3. This study was approved by the Institutional Review Boards at the North Carolina Department of Public Health, Communicable Disease Program and the University of North Carolina at Chapel Hill.

## Results

### Patient Characteristics

Between January 1, 1993 and December 31, 2006, a total of 6,416 verified cases of TB were reported and included in the North Carolina RVCT registry. Two hundred and ninety-two cases (4.6%) had both pulmonary and extrapulmonary disease and were excluded from analysis, resulting in a final study population of 6,124. Tables 1 and 2 show the distribution of socio-demographic and clinical variables by TB, HIV, and foreign birth status.

**Table 1:** Distribution of socio-demographic and clinical characteristics by disease site*.

**Socio-demographic and clinical characteristics**	**All TB cases (%) N = 6,124**	**EPTB (%) N = 1,366**	**PTB (%) N = 4,758**
**Verification^†^**			
Laboratory	5,143 (84.0)	1.057 (77.4)	4,086 (85.9)
Clinically	742 (12.1)	163 (11.9)	579 (12.2)
Provider diagnosis	239 (3.9)	146 (10.7)	93 (2.0)
**HIV status**			
Positive	563 (9.2)	151 (11.1)	412 (8.7)
Negative	3,388 (55.3)	757 (55.4)	2,631 (55.3)
Not tested	2,173 (35.5)	458 (33.5)	1,715 (36.0)
**Foreign-birth status**			
Foreign	1,299 (21.2)	402 (29.4)	897 (18.9)
United States	4,821 (78.7)	964 (70.6)	3,857 (81.1)
Missing	4 (0.1)	0 (0.0)	4 (0.1)
**Sex**			
Female	2,123 (34.7)	633 (46.3)	1,490 (31.3)
Male	4,001 (65.3)	733 (53.7)	3,268 (68.7)
**Time in US^‡^**			
0 – 1 years	384 (29.6)	84 (20.9)	300 (33.4)
2 – 5 years	446 (34.3)	136 (33.8)	310 (34.6)
>5 years	443 (34.1)	174 (43.3)	269 (30.0)
Missing	26 (2.0)	8 (2.0)	18 (2.0)
**Age (years)**			
0 – 4	150 (2.5)	29 (2.1)	121 (2.5)
5 – 14	136 (2.2)	33 (2.4)	103 (2.2)
15 – 24	422 (6.9)	112 (8.2)	310 (6.5)
25 – 44	1,872 (30.6)	459 (33.6)	1,413 (29.7)
45 – 64	1,753 (28.6)	334 (24.5)	1,419 (29.8)
65+	1,790 (39.23)	399 (29,2)	1,391 (29.2)
Missing	1 (0.0)		1 (0.0)
**Previous TB disease**			
Yes	334 (5.5)	70 (5.1)	264 (5.6)
No	5,783 (94.4)	1,293 (94.7)	4,490 (94.4)
Missing	7 (0.1)	3 (0.2)	4 (0.1)
**Homeless**			
Yes	410 (6.7)	35 (2.6)	375 (7.9)
No	5,681 (92.8)	1,321 (96.7)	4,360 (91.6)
Missing	33 (0.5)	10 (0.7)	23 (0.5)
**Drug use (injecting)**			
Yes	98 (1.6)	16 (1.2)	82 (1.7)
No	5,255 (85.8)	1,205 (88.2)	4,050 (85.1)
Missing	771 (12.6)	145 (10.6)	626 (13.2)
**Drug use (non-injecting)**			
Yes	580 (9.5)	71 (5.2)	509 (10.7)
No	4,785 (78.1)	1,150 (84.2)	3,635 (76.4)
Missing	759 (12.4)	145 (10.6)	614 (12.9)
**Excess alcohol use**			
Yes	1,255 (20.5)	124 (9.1)	1,131 (23.8)
No	4,137 (67.6)	1,104 (80.8)	3,033 (63.8)
Missing	732 (12.0)	138 (10.1)	594 (12.5)

* Excluding 292 (4.6%) with concomitant pulmonary and extrapulmonary disease.

^† ^Laboratory: positive *M. tuberculosis *culture or positive acid-fast stain; Clinical: positive tuberculin skin test, signs and symptoms consistent with TB, treatment with TB medication, and a completed diagnostic evaluation; Provider: cases that do not meet the laboratory or clinical case definition, but have a clinical picture consistent with TB.

^‡ ^Time in US prior to diagnosis among foreign born cases (n = 1,299).

**Table 2:** Socio-demographic and clinical characteristics by HIV and foreign birth status*.

**Socio-demographic and clinical characteristics**	**HIV+ (%) N = 563**	**HIV – (%) N = 3,388**	**Foreign Born (%) N = 1,299**	**US Born (%) N = 4,821**
**Verification^†^**				
Laboratory	516 (91.7)	2,873 (84.8)	1,070 (82.4)	4,070 (84.4)
Clinical	16 (2.8)	383 (11.3)	163 (12.6)	579 (12.0)
Provider	31 (5.5)	132 (3.9)	66 (5.1)	172 (3.6)
**Site of disease**				
Extrapulmonary	151 (26.8)	757 (22.3)	402 (31.0)	964 (20.0)
Pulmonary	412 (73.2)	2,631 (77.7)	897 (69.0)	3,857 (80.0)
**HIV status**				
Positive			74 (7.8)	471 (9.8)
Negative			943 (72.6)	2,444 (50.7)
Not tested			264 (20.32)	1,906 (39.5)
**Foreign-birth status**				
Foreign	92 (16.3)	943 (27.8)		
United States	471 (83.7)	2,444 (72.1)		
Missing	0 (0.0)	1 (0.0)		
**Time in US^‡^**				
0 – 1 years	22 (23.9)	271 (28.7)		
2 – 5 years	31 (33.7)	351 (37.2)		
>5 years	34 (37.0)	305 (32.3)		
Missing	5 (5.4)	16 (1.7)		
**Sex**				
Female	122 (21.7)	1,101 (32.5)	474 (36.5)	1,649 (34.2)
Male	441 (78.3)	2,287 (67.5)	825 (63.5)	3,172 (65.8)
**Age (years)**				
0 – 4	1 (0.2)	64 (1.9)	17 (1.3)	133 (2.8)
5 – 14	0 (0.0)	37 (1.1)	29 (2.2)	107 (2.2)
15 – 24	11 (2.0)	330 (9.7)	257 (19.8)	165 (3.4)
25 – 44	380 (67.5)	1,272 (37.5)	692 (53.3)	1,178 (24.4)
45 – 64	165 (29.3)	1,138 (33.6)	225 (17.3)	1,527 (31.7)
65+	6 (1.1)	546 (16.1)	78 (6.0)	1,711 (35.5)
Missing	1 (0.0)	0 (0.0)	1 (0.0)	0 (0.0)
**Previous TB disease**				
Yes	33 (5.9)	187 (5.5)	6 (5.1)	268 (5.6)
No	529 (94.0)	3,198 (94.4)	1,230 (94.7)	4,550 (94.4)
Missing	1 (0.2)	3 (0.1)	3 (0.2)	3 (0.1)
**Homeless**				
Yes	106 (18.8)	265 (7.8)	37 (2.9)	372 (7.7)
No	445 (79.0)	3,115 (92.0)	1,250 (96.2)	4,428 (91.9)
Missing	12 (2.1)	8 (0.2)	12 (0.9)	21 (0.4)
**Drug use (injecting)**				
Yes	48 (8.5)	42 (1.2)	6 (0.5)	92 (1.9)
No	432 (76.7)	3,069 (90.6)	1,229 (94.6)	4,024 (83.5)
Missing	83 (14.7)	277 (8.2)	64 (4.9)	705 (14.6)
**Drug use (non-injecting)**				
Yes	171 (30.4)	364 (10.7)	40 (3.1)	540 (11.2)
No	311 (55.2)	2,761 (81.5)	1,192 (91.8)	3,591 (74.5)
Missing	81 (14.4)	263 (7.8)	67 (5.2)	690 (14.3)
**Excess alcohol use**				
Yes	210 (37.3)	864 (25.5)	89 (6.9)	1,165 (24.2)
No	278 (49.4)	2,289 (67.6)	1,143 (88.0)	2,993 (62.1)
Missing	75 (13.2)	235 (6.9)	67 (5.2)	663 (13.8)

* Excluding 292 (4.6%) with concomitant pulmonary and extrapulmonary disease; among remaining TB cases, 2,173 (33.5%) were not tested for HIV and 4 (0.1%) were missing foreign birth status.

^† ^Laboratory: positive *M. tuberculosis *culture or positive acid-fast stain; Clinical: positive tuberculin skin test, signs and symptoms consistent with TB, treatment with TB medication, and a completed diagnostic evaluation; Provider: cases that do not meet the laboratory or clinical case definition, but have a clinical picture consistent with TB.

^‡ ^Time in US prior to diagnosis among foreign born cases (n = 1,299).

Extrapulmonary TB occurred in 1,366 (22.3%) TB patients. These cases were more often provider verified, HIV positive, foreign born, female, and 15 to 24 years old compared to PTB cases (Table 1). Among the foreign born, a larger proportion of EPTB cases had been in the US at least five years prior to TB diagnosis.

HIV co-infection was present in 563 (9.2%) of all TB cases, 35.5% had unknown HIV status due to testing not being performed. This decreased from 58.4% in 1993 to 8.1% in 2006. HIV co-infected TB cases were more often laboratory verified, diagnosed with EPTB, US born, male, 25 to 44 years old, with a recent history of being homeless and substance abuse in the past year (Table 2).

Foreign born cases accounted for 1,299 (21.2%) of all TB cases, increasing from 6.3% in 1993 to 37.2% in 2006. Over 60% came from 5 countries, including Mexico (38.0%), Vietnam (8.2%), India (6.9%), Philippines (4.4%), and Honduras (4.1%). Foreign born cases were more often diagnosed with EPTB, HIV negative, 15 to 44 years old, and lacked TB risk factors such as homelessness, illicit drug use, and excess alcohol use (Table 2). US born TB cases were primarily Black/African American (66.6%) or White/Caucasian (30.5%).

**Figure 2:**
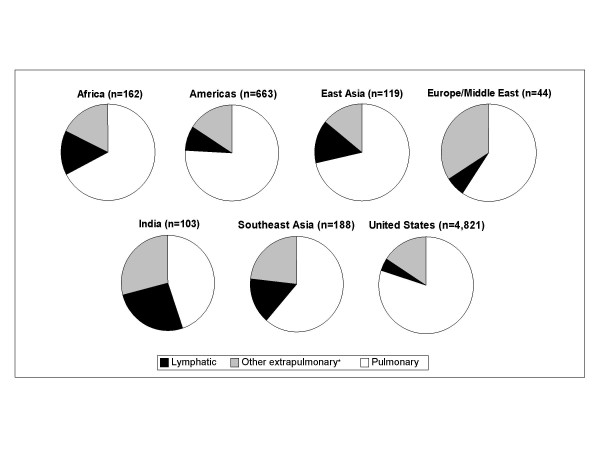
Distribution of disease site by region of birth. * Includes Bone/Joint, Genitourinary, Meningeal, Miliary, Peritoneal, Pleural and other non-specified sites.

The location of EPTB varied by HIV and foreign birth status (Table 3). The most common sites of disease were the pleura (25.1%) and cervical lymph nodes (15.7%). HIV co-infected individuals were more likely to have non-cervical lymph node or miliary disease compared to HIV uninfected individuals, while foreign born EPTB cases were more likely to have cervical lymphadenitis than US born cases. The site of EPTB also varied by region of birth (Figure 2). Lymphatic TB accounted for 50% of EPTB in East Asia, 47% in India, 45% in Africa, 40% in Southeast Asia, and 35% in the Americas, but only 17% in Europe/Middle East, and 21% in the US.

**Table 3:** Distribution of extrapulmonary disease by HIV status and foreign birth*.

**Extrapulmonary site**	**All EPTB cases (%) n = 1,366**	**HIV + (%) n = 151**	**HIV- (%) n = 757**	**P value^†^**	**Foreign born (%) n = 402**	**US born (%) n = 964**	**P value^†^**
Bone and/or joint	164 (12.0)	4 (2.7)	96 (12.7)	.003	48 (11.9)	116 (12.0)	.96
Genitourinary	78 (5.7)	5 (3.3)	40 (5.3)	.31	24 (6.0)	54 (5.6)	.79
Lymphatic: cervical	215 (15.7)	30 (19.9)	131 (17.3)	.45	119 (29.6)	96 (10.0)	<.001
Lymphatic: other	147 (10.8)	28 (18.5)	70 (9.3)	.008	41 (10.2)	106 (11.0)	.66
Meningeal	66 (4.8)	6 (4.0)	39 (5.2)	.54	21 (5.2)	45 (4.7)	.66
Miliary	158 (11.6)	38 (25.2)	87 (11.5)	<.001	35 (8.7)	123 (12.8)	.03
Other	135 (9.9)	15 (10.0)	73 (9.6)	.91	40 (10.0)	95 (9.9)	.96
Peritoneal	60 (4.4)	7 (4.6)	40 (5.3)	.74	12 (3.0)	48 (5.0)	.10
Pleural	343 (25.1)	18 (11.9)	181(23.9)	.001	62 (15.4)	281 (29.2)	<.001

* 458 (33.5%) of EPTB cases were missing HIV status.

^† ^Chi-square p-value for each category compared to all others

### Univariate and Multivariate Analysis

In a crude analysis comparing all foreign born with all US TB cases, we found that foreign born cases without HIV infection had a slightly stronger association with EPTB (OR 1.84; 95%; CI 1.55, 2.18) than US born cases with HIV infection (OR 1.34; 95% CI 1.06, 1.69). Those who were both foreign born and had HIV infection had nearly three times the odds of EPTB compared with HIV uninfected US born cases (OR 2.83; 95% CI 1.84, 4.34). Such generalizations, however, did not reflect the range of associations between EPTB and race/ethnicity, geographic regions, and HIV status.

Results of the detailed analysis are given in Table 4. After adjusting for year, sex, age, injecting drug use, and alcohol use, HIV uninfected foreign born cases were more likely to have EPTB with adjusted ORs ranging from 1.36 to 5.09, depending on the region of origin. Among US born cases, only Black/African American ethnicity was associated with EPTB in the absence of HIV infection (OR 1.84; 95% CI 1.42, 2.39).

**Table 4:** Odds ratios for each category of exposure and the association with EPTB.

	HIV Uninfected			HIV Infected		
Race/ethnicity, Region	N (%)	Crude OR (95% CI)*	Adj. OR (95% CI)*	N (%)	Crude OR (95% CI)*	Adj. OR (95% CI)*
United States White	696 (17.7)	Referent	Referent	43 (1.1)	2.36 (1.17, 4.76)	1.04 (0.38, 2.79)
United States Black	1,638 (41.7)	1.67 (1.31, 2.13)	1.84 (1.42, 2.39)	419 (10.7)	1.89 (1.38, 2.58)	2.60 (1.83, 3.71)
United States Hispanic	65 (1.7)	1.24 (0.63, 2.46)	1.18 (0.56, 2.48)	7 (0.2)	4.58 (1.01, 20.76)	3.30 (0.54, 20.19)
United States Other	40 (1.0)	1.08 (0.44, 2.63)	1.13 (0.45, 2.83)	2 (0.1)	n/a	n/a
						
Africa	123 (3.1)	3.28 (2.14, 5.03)	2.28 (1.45, 3.59)	21 (0.5)	1.44 (0.47, 4.36)	0.97 (0.32, 3.00)
Americas	482 (12.3)	1.72 (1.27, 2.33)	1.36 (0.97, 1.91)	64 (1.6)	5.73 (3.36, 9.79)	5.12 (2.84, 9.23)
East Asia	79 (2.0)	2.36 (1.38, 4.03)	1.62 (0.93, 2.80)	0 (0.0)	n/a	n/a
Europe/Middle East	28 (0.7)	4.58 (2.10, 9.97)	3.38 (1.47, 7.76)	0 (0.0)	n/a	n/a
India	75 (1.9)	7.77 (4.69, 12.85)	5.09 (3.01, 8.60)	0 (0.0)	n/a	n/a
Southeast Asia	147 (3.7)	3.98 (2.68, 5.90)	2.88 (1.90, 4.37)	2 (0.1)	6.10 (0.38, 98.36)	n/a

* OR, odds ratio; CI, confidence interval; Adj., adjusted for year, age, sex, injecting drug use, and alcohol use.

^† ^Includes Asian, Pacific Islander, Alaskan Native, and American Indian.

^‡ ^Cell counts too small to calculate association

HIV infection was also found to be associated with EPTB. Among US born cases, HIV infection was associated with EPTB in Black/African Americans (OR 2.60; 95% CI 1.83, 3.71). Among the foreign born, HIV infection was associated with EPTB among those from the Americas region (OR 5.12; 95% CI 2.84, 9.23). The association could not be assessed for 4 regions because of small numbers of HIV co-infected patients.

## Discussion

This study analyzed and quantified the individual and joint associations of HIV infection and foreign birth with EPTB while controlling for important confounders. We observed that foreign born TB cases were more likely to have exclusive EPTB than US born TB cases, even in the absence of HIV infection. HIV infection was also associated with exclusive EPTB above and beyond the effects of race/ethnicity or geographic region for Black/African Americans and foreign born cases from the Americas. We also found that the site of EPTB differed by region and HIV status. Lymphadenitis accounted for a disproportionate amount of EPTB among foreign born cases from East and Southeast Asian, Indian, and African regions. Miliary disease and non-cervical lymphadenitis were more common among HIV co-infected cases.

Others have also documented an association between EPTB and foreign birth. Wilbershied et al. observed ORs, adjusted for HIV infection and other covariates, ranging from 0.9 to 3.9 for the association of exclusive EPTB with foreign born populations in New York City [9]. They did not account for race/ethnicity among the US born cases, which is an important limitation given we found that the occurrence of EPTB may differ by race/ethnicity.

A study of EPTB in Canada by Yang et al. found similar associations between foreign born status and EPTB, with crude ORs ranging from 1.72 to 2.53, when compared to non-Aboriginal Canadians [17]. HIV status was not adjusted for as only 1.8% of TB cases had HIV co-infection. Patients with both pulmonary and extrapulmonary TB (8.3%) were included in the pulmonary TB group as opposed to being excluded as in our study.

Ong et al. reported an OR of 1.62 for the association of foreign birth with exclusive EPTB among TB cases in San Francisco [8], but the finding was not statistically significant. Their findings were adjusted for HIV status and race/ethnicity, but did not account for region of birth among foreign born TB cases.

Similar to our findings, others have observed an association between EPTB and HIV. Ong et al. and Wilberschied et al. found small associations, with ORs of 1.3 to 1.45 while adjusting for foreign birth [8,9]. Onorato et al. reported that HIV infected persons were twice as likely to have EPTB as HIV uninfected persons [11], and Yang et al. found that those with HIV infection had nearly 5 times the odds of EPTB than HIV uninfected TB cases [10]. These last two studies, however, included cases with concomitant PTB in the EPTB category. Concomitant pulmonary and extrapulmonary disease has been shown to be more common in HIV infected persons [7], which may explain the stronger association they observed.

While EPTB among HIV infected persons is related to immunosuppression, little is known about the factors associated with EPTB among foreign born populations. Some authors have speculated that this is an artifact of screening immigrants for PTB, which could inflate the proportion of EPTB by removing or treating those with PTB prior to immigration [18,19]. We feel this is unlikely, as such an affect would be seen mainly in the first year of immigration, while our and other data [9] suggest that EPTB occurs more often among immigrants who have been in the US more than 5 years. Other factors such as genetic variations in *Mycobacterium tuberculosis *and/or genetic variations in host immune response may account for the observations. Yang et al. found that TB patients infected with *M. tuberculosis *(MTB) containing a mutation in the phospholipase-C gene D had more than two times the odds of having extrathoracic TB (with or without thoracic involvement) compared to those with the wild type strain (OR 2.19; 95% CI 1.27, 3.76), while controlling for HIV infection, sex and race [20]. Other studies have found genetic polymorphisms in host immune responses that are associated with EPTB, including Manose-Binding Protein and TB meningitis [21], Interleuken (IL)-1β/IL-1R [22], IL-10 and IFN-γ [23], and NRAMP1 and pleural TB [24]. Fernando et al. found an association between a polymorphism in the P2X_7 _gene and EPTB [25]. P2X_7 _is a receptor expressed on macrophages which facilitates induction and death of MTB. Polymorphisms that inhibit receptor activity may facilitate the spread of MTB to extrapulmonary sites. Among Southeast Asian refugees in Australia, those with EPTB were more likely to have the polymorphism than healthy controls or patients with pulmonary TB (OR 3.8; 95% CI 1.6, 9.0).

**Figure 3:**
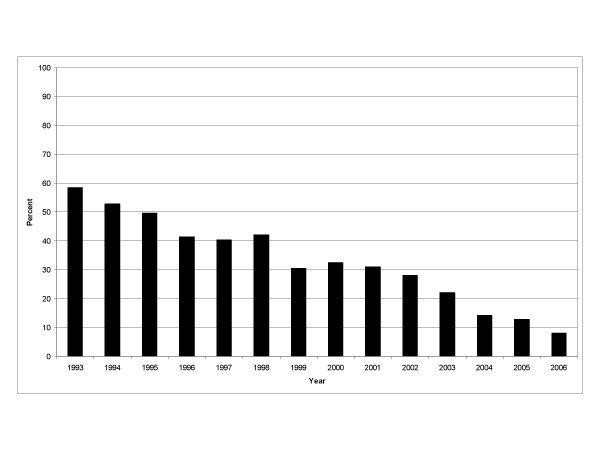
Decline in percent of TB patients not tested for HIV infection, North Carolina, 1993–2006.

Potential limitations of our study should be acknowledged. HIV testing was not done for 35.5% of the reported TB cases in our study. This has been noted in other studies analyzing surveillance data, with 30% to 50% of TB patients having unknown HIV status [9,10,26]. However, this is unlikely to affect validity and reliability of the data as noted in a recent analysis of California's TB registry [27]. While HIV testing and counseling is offered free of charge to TB patients at the health departments, incomplete implementation of routine HIV testing likely accounts for why many patients were not tested. The patient may not have been offered an HIV test due to perceived low risk by the clinician, or the patient may have refused testing when offered. Either of these scenarios would result in missing HIV data. In our study, 54% of patients not tested for HIV were at low risk for HIV (>44 years old with no reported substance abuse or homelessness). Nevertheless, testing improved during the study period with 92% of TB cases reported in 2006 receiving HIV testing (Figure 3).

Small numbers of HIV co-infected TB patients hindered our ability to analyze EPTB among foreign born persons with HIV infection, and the absence of CD4 count data precluded a more detailed analysis by level of immunosupression. This may be important as some studies have shown that EPTB, especially disseminated or meningeal disease, occurs more often in individuals with cell counts less than 200 cells/μl [28-30], even though other studies found no association between frequency of EPTB and decreasing CD4+ cell counts [31-33].

## Conclusion

Extrapulmonary TB poses an important hurdle for the elimination of TB in the US. The proportion of EPTB will likely increase as foreign born and HIV attributable TB cases continue to rise in the US. Morbidity and mortality may be exacerbated in this group because of stigma, language, cultural, or immigration-related barriers to timely healthcare. Further research is needed to explore why the occurrence and type of EPTB differs by region of birth and whether host genetic and/or bacterial variation can explain these differences in EPTB.

## Competing interests

The author(s) declare that they have no competing interests.

## Authors' contributions

AMK is responsible for data analysis and writing the manuscript. CDH is responsible for acquisition of the data. All authors contributed to the design of the study and critical review of the manuscript.

## Appendix: Countries of origin and the corresponding region for all Tuberculosis cases used in the analysis

**African: **Algeria, Benin, Burkina, Cameroon, Chad, Comoros, Congo, Democratic Republic of Congo, Djibouti, Egypt, Ethiopia, Europa Island, Gabon, Gambia, Ivory Coast, Kenya, Liberia, Mauritius, Mayotte, Morocco, Niger, Nigeria, Reunion, Senegal, Sierra Leone, Somalia, South Africa, Sudan, Togo, Uganda, Zambia.

**American: **Argentina, Barbados, Brazil, Cayman Islands, Colombia, Costa Rica, Cuba, Dominican Republic, Ecuador, El Salvador, Guadeloupe, Guatemala, Haiti, Honduras, Mexico, Nicaragua, Panama, Peru, Trinidad and Tobago, Uruguay, Venezuela.

**East Asian: **China, Hong Kong, Japan, North and South Korea, Macau, Nauru, Philippines, Pitcairn Islands, Taiwan.

**European/Middle Eastern: **Albania, Azerbaijan, Belarus, Bosnia and Hercegovina, France, Georgia, Germany, Greece, Iran, Ireland, Kazakhstan, Kuwait, Monaco, Oman, Romania, San Marino, Slovenia, Soviet Union, Spain, Syria, Turkey, Ukraine, United Arab Emirates.

**Indian: **India, Pakistan.

**Southeast Asian: **Bangladesh, Burma, Cambodia, Indonesia, Laos, Malaysia, Nepal, Thailand, Vietnam.

**United States: **United States of America.

## Pre-publication history

The pre-publication history for this paper can be accessed here:


